# Immunohistochemical characterization of interstitial cells and their relationship to motor neurons within the mouse esophagus

**DOI:** 10.21203/rs.3.rs-4474290/v1

**Published:** 2024-06-11

**Authors:** Emer Ni Bhraonain, Jack Turner, Karen Hannigan, Kenton Sanders, Caroline Cobine

**Affiliations:** University of Nevada Reno; University of Nevada Reno; University of Nevada Reno; University of Nevada Reno; University of Nevada Reno

**Keywords:** Interstitial cells of Cajal, PDGFRα+ cells, neurons, glia, motility

## Abstract

Interstitial cells of Cajal (ICC) and PDGFRα^+^ cells regulate smooth muscle motility in the gastrointestinal (GI) tract. However, their role(s) in esophageal motility are still unclear. The mouse esophagus has traditionally been described as almost entirely skeletal muscle in nature though ICC have been identified along its entire length. The current study evaluated the distribution of skeletal and smooth muscle within the esophagus using a mouse selectively expressing eGFP in smooth muscle cells (SMCs). The relationship of SMCs to ICC and PDGFRα^+^ cells was also examined. SMCs declined in density in the oral direction however SMCs represented ~ 25% of the area in the distal esophagus suggesting a likeness to the transition zone observed in humans. ANO1^+^ intramuscular ICC (ICC-IM) were distributed along the length of the esophagus though like SMCs, declined proximally. ICC-IM were closely associated with SMCs but were also found in regions devoid of SMCs. Intramuscular and submucosal PDGFRα^+^ cells were densely distributed throughout the esophagus though only intramuscular PDGFRα^+^ cells within the LES and distal esophagus highly expressed SK3. ICC-IM and PDGFRα^+^ cells were closely associated with nNOS^+^, VIP^+^, VAChT^+^ and TH^+^ neurons throughout the LES and distal esophagus. GFAP^+^ cells resembling intramuscular enteric glia were observed within the muscle and were closely associated with ICC-IM and PDGFRα^+^ cells, occupying a similar location to c. These data suggest that the mouse esophagus is more similar to the human than thought previously and thus set the foundation for future functional and molecular studies using transgenic mice.

## Introduction

The esophagus transports food, liquids and saliva from the oral cavity to the stomach. This movement occurs via highly coordinated peristaltic waves. The mechanisms underlying contractile activity within the esophagus are complex in that it is composed of both skeletal and smooth muscle with differences in composition occurring between species. In the human esophagus the proximal one third is skeletal muscle, the distal one third is smooth muscle and the middle one third represents a transition from skeletal to smooth muscle (Meyer et al., 1986). Primary peristalsis occurs in the proximal esophagus where somatic neural inputs regulate contraction of the skeletal muscle. In contrast, secondary peristalsis occurs where smooth muscle is located and is initiated in part by luminal distention (Nikaki et al., 2019).

Peristalsis also occurs in the small and large intestines where enteric neurons play an important role in regulating the movement of luminal contents. In addition to enteric neural inputs, interstitial cells of Cajal (ICC) and platelet-derived growth factor receptor alpha-positive cells (PDGFRα^+^ cells) have been shown to regulate motility (Sanders et al., 2024, Sanders et al., 2014). ICC play an important role as pacemaker cells and as mediators of cholinergic and nitrergic neurotransmission (Burns et al., 1996, Drumm et al., 2019, Duffy et al., 2012, Hannigan et al., 2024, Sung et al., 2018, Ward et al., 2000). These cells highly express the Ca^2+^-activated Cl^−^ channel ANO1, a conductance underlying slow wave activity and responses to neurotransmission (Cobine et al., 2017, Hwang et al., 2009, Sung et al., 2018, Zhu et al., 2009). PDGFRα^+^ cells mediate purinergic responses in the colon and stomach (Baker et al., 2013, Kurahashi et al., 2011) and express P2Y_1_ receptors (P2Y_1_R) and small conductance Ca^2+^-activated K^+^ channels (SK3) (Hannigan et al., 2024, Kurahashi et al., 2011, Peri et al., 2013). PDGFRα^+^ cells also express α1 adrenoceptors (Ha et al., 2017, Kurahashi et al., 2020) and pituitary adenylate cyclase-activating polypeptide (PACAP) receptors (PAC_1_R) (Ha et al., 2017, Kurahashi et al., 2022).

Previous studies evaluating the distribution of ICC in the esophagus of the dog, guinea-pig and mouse noted the presence of ICC within the skeletal muscle region (Berezin et al., 1994, Burns et al., 1997, Daniel and Posey-Daniel, 1984, Rumessen et al., 2001) albeit their density was much lower than in the smooth muscle of the adjacent lower esophageal sphincter (LES). Additional studies have characterized the distribution of inhibitory nitrergic and excitatory cholinergic nerve fibers within the guinea-pig esophagus, and similar to the distribution of ICC, the greatest density of these nerve fibers was within the LES and declined proximally (Brookes et al., 1996). Despite these early observations, the function of ICC has not been evaluated within this region and therefore their role within the skeletal muscle is still unclear. Studies in patients with achalasia, a disease associated with disrupted peristalsis and impaired relaxation of the LES have noted a decrease in density of both ICC and nitrergic neurons (Gockel et al., 2008, Qian et al., 2023) while others have suggested that the association of ICC and nitrergic neurons is reduced in achalasic patients, rather than their density (Chen et al., 2013). PDGFRα^+^ cells and S100β^+^ cells are also found within the human esophagus (Qian et al., 2023). Though the morphology, distribution and function of ICC and PDGFRα^+^ cells and their relationship to enteric neurons have been characterized throughout most of the length of the gastrointestinal (GI) tract, their distribution and morphological relationships with other cells has not been characterized in as much depth within the LES and esophagus. Therefore, the main focus of the current study was to evaluate the morphology and distribution of ICC, PDGFRα^+^ cells, neurons and glia, and their spatial relationship to one another within the mouse esophagus and LES. These studies will likely provide critical insight for future functional studies particularly given the availability of transgenic mouse models. This in turn may provide better understanding of the physiology of the human esophagus and aid in obtaining greater understanding of what occurs in disease.

## Materials and methods

### Animals

All procedures were approved by the Institutional Animal Care and Use Committee at the University of Nevada, Reno. Animals used and experiments performed were also in accordance with the National Institutes of Health Guide for the Care and Use of Laboratory Animals. Adult C57BL/6 mice (wildtype, WT, strain number: 000664, RRID: IMSR_JAX:000664) and SmMHC^eGFP/+^ mice (Strain number: 007742 RRID: IMSR_JAX:007742) of both sexes were used in this study. The animals were euthanized with isoflurane (Baxter, Deerfield, IL, USA) followed by cervical dislocation.

### Tissue preparation

The stomach and esophagus were removed *en bloc* and placed in a Sylgard-lined dissection dish with ice cold Krebs-Ringer bicarbonate solution (KRBS; composition (in mM): 118 NaCl, 4.7 KCl, 2.5, CaCl_2_, 1.2 MgCl_2_, 23.8 NaHCO_3_, 1.2 KH_2_PO_4_, 11 dextrose; pH of 7.4 after bubbling to equilibrium with 95% O_2_/5% CO_2_). The esophageal body was opened by following the lesser curvature of the stomach. The esophagus and LES clasp muscle were pinned fiat and the serosa and mucosa were removed with sharp dissection.

### Isometric tension recording

Strips of circular muscle measuring 1 mm in width were taken from the LES or distal esophagus and attached to a force transducer and stable mount with suture. Tissues were immersed in oxygenated KRBS in a tissue bath heated to 37°C and the LES and distal esophagus were stretched to 0.25 grams and 1 gram respectively. All experiments were done in the presence of tetrodotoxin (TTX) (1 μM, Cat. # 14964, Cayman Chemical, Ann Arbor, MI, USA; RRID:SCR_008945) to isolate the myogenic component and remove the influence of neural inputs. Data was recorded using AcqKnowledge 3.9.1 software (Biopac Systems Inc; RRID:SCR_014829).

### Tissue preparation for whole-mount immunohistochemistry

The esophagus and stomach were removed and dissected in ice cold KRBS as described above. The esophageal body was opened by following the lesser curvature of the stomach. The esophagus and LES clasp muscle were pinned flat and the serosa and mucosa were removed with sharp dissection. The flat sheet muscle preparation was transferred to a Sylgard-lined dish and pinned out with the circular muscle facing upwards. For immunohistochemistry, tissues were fixed in the dish for 15 minutes in 4% paraformaldehyde (PFA) at 4°C.

### Tissue preparation for cryosectioning

For cryosectioning, the esophagus and LES were kept intact in a tube and sharp dissection was used to remove the serosal layer. For fixation, a 1 mm diameter glass capillary rod was inserted through the lumen of the esophagus and tissue was loosely pinned to anchor it in a Sylgard-lined dish where it was fixed for 15 minutes in 4% PFA at 4°C. Tissues were then washed in 0.01 M PBS six times for 15 minutes, dehydrated through graded concentrations of sucrose solution (5%, 10%, 15%, 20%), and left in 20% sucrose solution overnight at 4°C. Tissues were embedded as an intact tube in a solution of 20% sucrose and Tissue Tek OCT compound (Sakura Finetek, Torrance, CA, USA) (1:1) and stored in a −80°C freezer until sectioning. 16 pm thick sections were cut parallel to the circular muscle layer using a Leica CM 3050 cryostat (Leica Microsystems, Wetzlar, Germany; RRID:SCR_020214). Sections were air dried for two hours at room temperature and immersed in 0.01 M PBS to remove excess OCT.

### Immunohistochemical labeling

Following fixation or sectioning tissues/slides were washed in 0.01 M PBS six times for 15 minutes. To ensure adequate permeabilization of primary antibodies, tissues/slides were blocked in a buffer containing 0.25% Triton-X (Sigma-Aldrich, St Louis, MO, USA; RRID:SCR_008988) and 0.5% BSA (Sigma-Aldrich, St Louis, MO, USA; RRID:SCR_008988) for two hours, at room temperature. Primary antibodies (Table 1) were diluted in 0.5% Triton-X and tissues were subsequently incubated for 48 hours (tissues) or overnight (slides) at 4°C in the fridge. Tissues/slides were washed in 0.01 M PBS solution six times for 15 minutes each. Incubation in the appropriate secondary antibodies (Table 1) was carried out for 1 hour at room temperature in the dark followed by six 15 minute washes in 0.01 M PBS. If dual labeling was carried out, tissues/slides were blocked again in 0.25% Triton X and 0.5% BSA and placed into the second primary antibody diluted under the same conditions described above. Tissues/slides were washed in 0.01 M PBS solution six times for 15 minutes each. Incubation in the appropriate secondary antibodies (Table 1) was carried out for 1 hour at room temperature in the dark followed by six 15 minute washes in 0.01 M PBS. Tissues and slides were mounted using Vectashield antifade mounting media (Vector Laboratories, Newark, CA, USA; RRID:SCR_000821).

### Imaging of antibody labeling

All imaging was done using a Leica Stellaris 5 HyD S Confocal Microscope (Leica Microsystems, Morrisville, NC, USA; RRID:SCR_024663) at a resolution of 1024×1024 dpi. Laser power, gain, offset and voltage were system optimized and kept consistent between all samples. Images are digital composites of Z-series with the exception of images in Fig. 3 which are mosaic merges across one imaging plane. Individual image conditions are outlined in Supplemental table 1. Final images were constructed using LasX software (Leica Microsystems, Morrisville, NC, USA; RRID:SCR_013673) and CorelDRAW 2023 (Alludo, Ottawa, ON, Canada; RRID:SCR_014235).

### Cell counting

Immunohistochemical labeling was carried out on whole-mount esophagus and LES preparations using a Kit antibody to visualize ICC. Images were obtained from each millimeter distance along the entire length of the mouse esophagus (1–30 mm) (n = 5) and Kit^+^ cells were counted at each length. Kit^+^ cells were defined as spindle-shaped entities with a visible cell body and robust fluorescent labeling, and cell count was normalized as a percentage of Kit^+^ cells in the LES.

### Pixel Analysis

Pixel analysis to quantify the mean area occupied by red pixels (skeletal muscle) or green pixels (smooth muscle) was done using Fiji (version 2.9, National Institutes of Health, MD, USA; RRID:SCR_002285). Image volume channels for red and green were thresholded to generate 3D binary masks (Supplemental Table 2) and fractional volumes were calculated using the Analyze Particles Macro.

## Results

### Gross anatomy of the mouse esophagus

**Figure 1:**
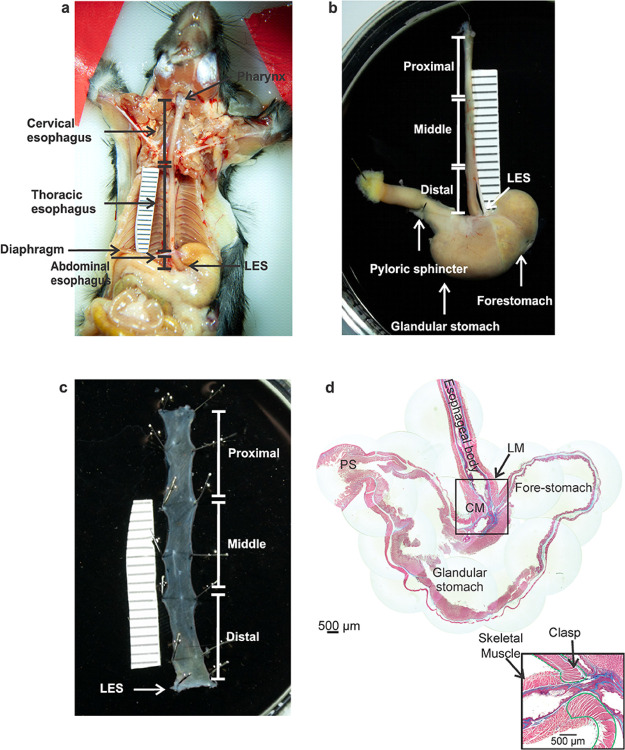
Gross Anatomy of the mouse esophagus and gastroesophageal junction **a**Anatomy of the mouse esophagus *in situ*, demonstrates the cervical, thoracic and abdominal portions of the intact esophageal body, image shows the esophagus traversing the diaphragm to join the stomach at the gastroesophageal region. **b**Removal of the esophagus and stomach from the mouse for dissection. **c** the esophagus is pinned out as a flat sheet with LES intact, the boundaries of proximal, middle and distal esophagus are indicated and the LES clasp is identified with a white arrow. **d** Trichrome staining of sagittal sections of the intact mouse esophagus and stomach show longitudinal muscle layer (LM), and circular muscle layer (CM) (pink) of the esophagus arranged perpendicular to one another, the LES clasp muscle is identified by the green dashed line at the gastroesophageal junction (adapted from (Drumm et al., 2022)).

The entire esophagus from pharynx to stomach was evaluated *in situ in* euthanized mice. The esophagus varied in length from 31 to 35 mm (n = 6) (Table 2b) coursing through the cervical, thoracic and abdominal body cavities (Fig. 1a). The cervical portion of the esophagus begins at the pharynx and ends at the sternum, measuring approximately 14 mm in length. The thoracic esophagus extends from the sternum to the diaphragm measuring approximately 16 mm. The abdominal esophagus begins at the hiatal diaphragm and ends with the LES, a thickening of the circular smooth muscle at the gastroesophageal junction (GEJ). This latter portion is the shortest segment, measuring approximately 3 mm in length. For this study, the intact esophagus and stomach were removed from the mouse *en bloc*. Skeletal muscle was observed along the majority of the esophagus, transitioning to smooth muscle approximately 1 mm proximal to where it joined with the stomach. Differences in muscle type were noted based on differences in coloration with the skeletal muscle being darker than smooth muscle (Fig. 1b, c). The esophagus was divided into proximal, middle and distal segments, each 10–12 mm in length. The LES clasp muscles located at the termination of the esophagus, were visualized as a band of whiteish muscle fibers oriented in the circumferential direction (Fig. 1c). Images of trichrome staining on sagittal sections of the esophagus and stomach from our previously published study (Drumm et al., 2022) demonstrated the distribution of muscle (pink) and connective tissue (blue) in the esophageal body and LES (Fig. 1d). The esophageal body consists of a thick circular muscle (CM) layer measuring 137.62 ± 47 μm (n = 3) in diameter and a slightly thinner longitudinal muscle (LM) layer measuring 108.7 ± 39 μm (n = 3), arranged perpendicular to one another (Table 2a). The LES (identified with the dashed green line (Fig. 1d)) had the thickest diameter of circular muscle, measuring 619.47 ± 69.5 μm (n = 3, Table 2a).

### Comparison of contractile activity in the LES and distal esophagus

**Figure 2:**
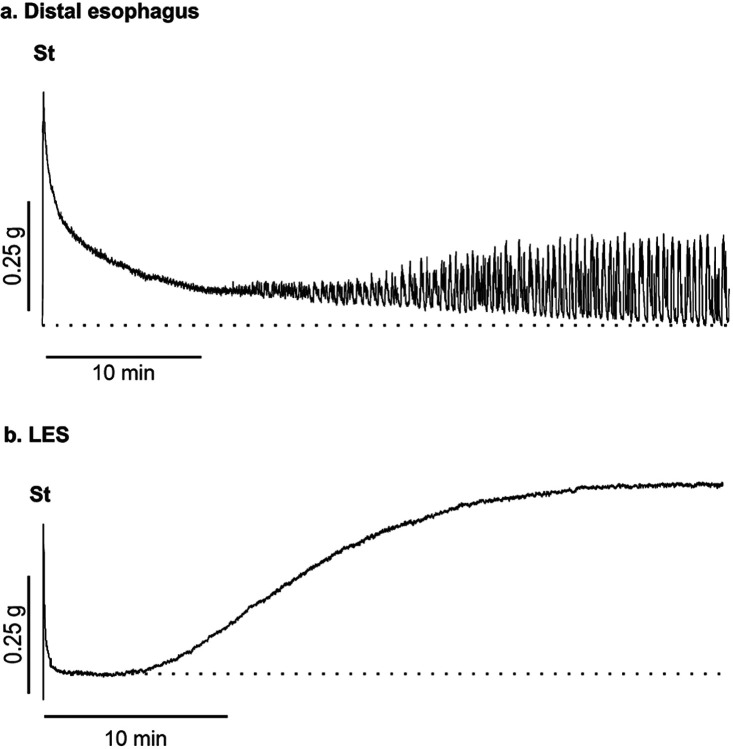
Contractile activities differ in the distal esophagus and LES Isometric tension recordings were carried out on strips of distal esophagus and LES circular muscle (TTX present throughout). Example traces of contractile activity are shown. The baseline is indicated by a dotted line. **a** The distal esophagus was stretched by 1 g (st) and after an equilibration period generated rhythmic phasic contractions that persisted in the presence of TTX. **b**The LES was stretched to 0.25 g (st) and after a short equilibration period generated a sustained contraction or ‘tone’ and no phasic activity. This activity was also insensitive to TTX.

Strips of LES and distal esophagus circular muscle were used in isometric tension recordings to compare contractile activity between these two regions. The distal esophagus generated rhythmic phasic contractions (Fig. 2a, n = 5), whereas as observed in our previous study (Drumm et al., 2022), the LES generated tone (Fig. 2b, n = 5). This activity was myogenic in nature as it occurred in the presence of the neural blocker tetrodotoxin (TTX, 1 μM). Despite differing contractile patterns, it is likely that both muscle regions in tandem with the crural diaphragm contribute to the generation of a high-pressure zone at the GEJ (Vegesna et al., 2013).

### Characterization of smooth muscle cells in the mouse esophagus

**Figure 3:**
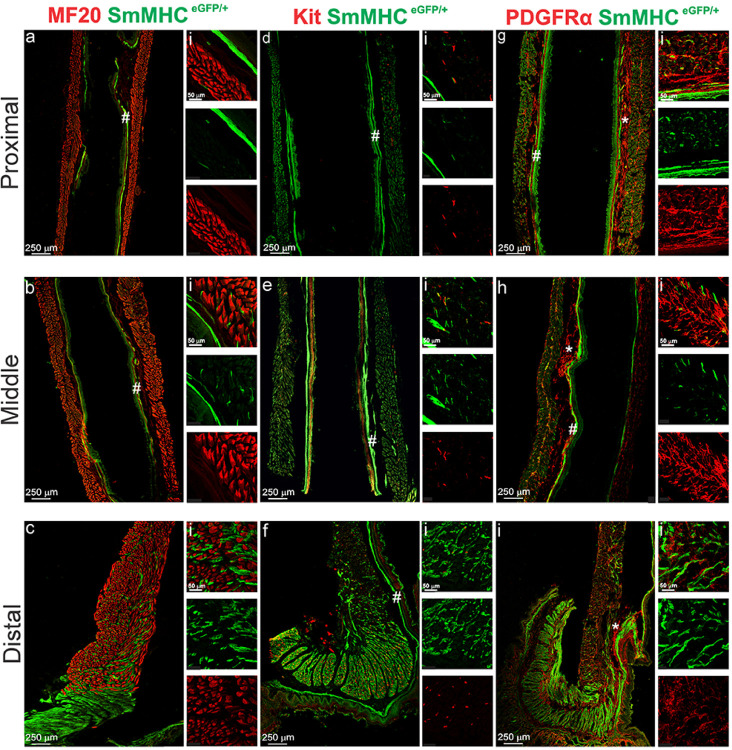
SMCs, ICC, and PDGFRα^+^ cells are present throughout the mouse LES and esophagus, and form close contacts with one another Immunohistochemistry on cryosections of SmMHC/^eGFP+^ mouse esophagus shows presence of SMCs between skeletal muscle fibers in the proximal **a**, middle **b** and distal esophagus **c** as well as in a distinct band located beneath the mucosa (muscularis mucosae). Panels **d-f** show the presence of Kit^+^ ICC-IM within SMC bundles and in close proximity to skeletal muscle fibers in the proximal **d**, middle **e** and distal esophagus **f**, the density of ICC-IM was greatest in the LES clasp and distal esophagus f, but this declined in density proximally **d-e**. Panels **g-i** show two populations of PDGFRα^+^ cells, one located within the muscle (intramuscular) and another that forms a network in the submucosal region. These cells were located within SMC bundles and alongside skeletal muscle fibers in the proximal **g**, middle **h** and distal esophagus **i**. The density of both intramuscular and submucosal PDGFRα^+^ cells remained constant throughout the esophagus. Smaller panels indicated by i are higher magnification images of the panels to their left. (* = submucosa, # = muscularis mucosae)

While skeletal muscle is apparent throughout the mouse esophageal body (Fig. 1, Fig. 3a–c), and the distal esophagus generates phasic contractions (Fig. 2a), it has not yet been determined as to whether smooth muscle is also present within the esophagus. To address this question, we examined cryosections taken from mice selectively expressing eGFP in smooth muscle cells (SMCs) (SmMHC^eGFP/+^ mice) and labeled with an antibody against GFP. In the LES clasp, smooth muscle was divided into a robust CM layer and a less apparent LM layer. The CM was divided into ~ 5–7 smooth muscle bundles (Fig. 1d, Fig. 3c,f,i). A distinct smooth muscle layer associated with the mucosa, i.e., the muscularis mucosae, was also observed in this region extending from the stomach to the proximal esophagus (Fig. 3a–i). The density of SMCs within the CM and LM decreased in the proximal direction. In the section of distal esophagus shown, approximately 24.3% (n = 1) (Fig. 3c) of the tissue volume was occupied by SMCs compared to only 5.14% in the middle esophagus (n = 1) (Fig. 3b, Supplemental Table 2). In contrast, only occasional single SMCs were observed in the proximal esophagus and these were present within skeletal muscle bundles and occupied roughly 2.3% of the tissue (n = 1) (Fig. 3a, Supplemental Table 2).

### Characterization of interstitial cells of Cajal (ICC)

**Figure 4:**
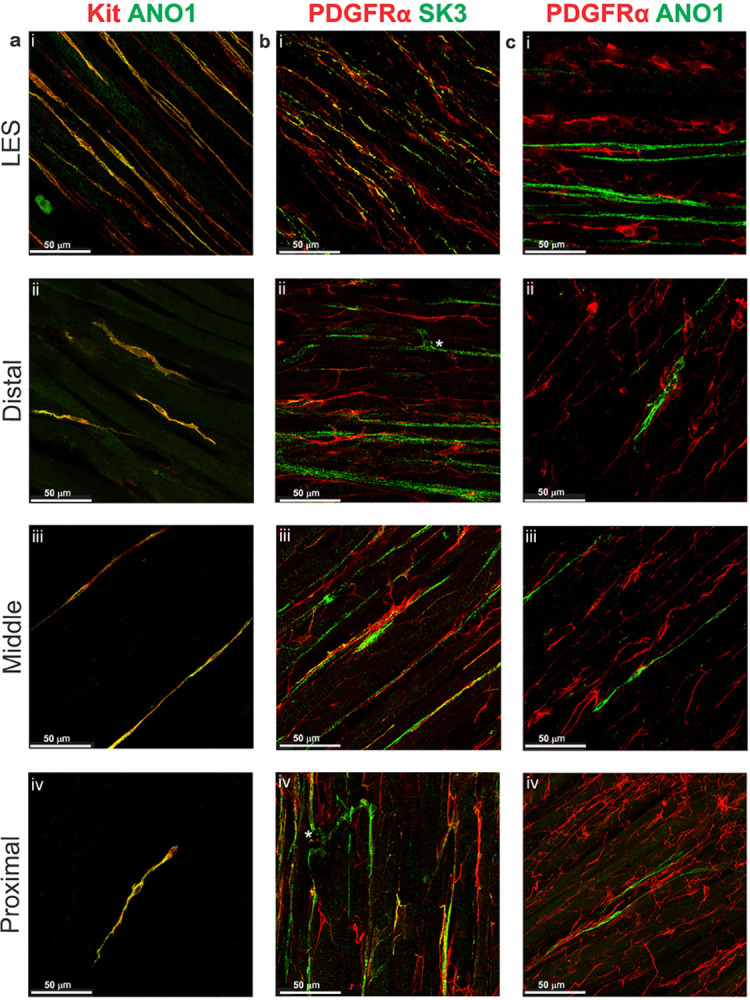
ICC-IM and PDGFRα^+^-IM cells are closely associated throughout the esophagus and LES **a** Dual IHC labeling of whole-mount mouse esophagus and LES with antibodies against Kit (red) and the Ca^2+^ activated Cl” channel, ANO1 (green) revealed that ICC-IM highly express ANO1 in the mouse LES (i), distal (ii), middle (iii) and proximal esophagus (iv). **b** Dual IHC labeling of whole-mount mouse esophagus and LES with antibodies against PDGFRα (red) and the small conductance Ca^2+^ activated K^+^ channel, SK3 (green) revealed that PDGFRα^+^-IM in the LES clasp highly express SK3 channels (i). Occasionally PDGFRα^+^ cells in the distal (ii), middle (iii) and proximal esophagus (iv) did express SK3 however this was less common than in the LES clasp, instead SK3 expression was noted in vascular like structures (indicated by white asterisks *). **c** ANO1 (green) and PDGFRα (red) dual labeling shows a close association between ANO1^+^ ICC-IM and PDGFRα-IM in the mouse LES (i), distal esophagus (ii), middle esophagus (iii) and proximal esophagus (iv).

Cryosections from the same preparations of SmMHC^eGFP/+^ mouse LES and esophagus were labeled with antibodies against GFP and Kit to evaluate the morphology and distribution of ICC and SMCs (Fig. 3d–f). In the LES clasp, ICC were present within each CM bundle (intramuscular ICC; ICC-IM) running parallel to SMCs (Fig. 3f, Supplemental Fig. 1ai). Notably, ICC were absent from the myenteric and submucosal regions of the LES clasp. In the esophagus ICC-IM were present within CM and LM bundles and similar to the LES clasp, no ICC were present in either the myenteric or submucosal regions. In the distal esophagus where the smooth muscle transitioned to skeletal muscle, the density of ICC-IM decreased in line with the decreased density of SMCs (Fig. 3f, Supplemental Fig. 1b). The greatest density of ICC-IM within the esophagus was at 4 mm proximal to the LES, (49.4%, n = 5) this is consistent with where the sling fibers of the LES converge in the esophagus. The density of ICC-IM decreased further in the middle and proximal esophagus, with only occasional ICC-IM being observed within the proximal esophagus (Fig. 3d, e, Supplemental Fig. 1b). In most cases, ICC-IM were closely associated with SMCs but on occasion they were noted in regions occupied exclusively by skeletal muscle (Fig. 3d,e,f). Additional immunohistochemical (IHC) studies were performed on whole-mount preparations from wildtype mice to evaluate the expression of ANO1. ICC in other regions of the GI tract including the LES clasp express ANO1 (Cobine et al., 2017, Drumm et al., 2022, Gomez-Pinilla et al., 2009, Hwang et al., 2009). Kit^+^ ICC-IM were spindle-shaped in morphology (Supplemental Fig. 1a) and robust ANO1 labeling was present in all ICC-IM throughout the LES clasp, distal, middle and proximal esophagus (Fig. 4ai–iv) suggesting that these cells may share a similar functional role to ICC in other GI muscles.

### Characterization of PDGFRα^+^ cells

The distribution of PDGFRα^+^ cells was evaluated in a manner similar to that described above for ICC. Cryosections from the same preparations of SmMHC^eGFP/+^ mouse LES and esophagus were labeled with antibodies against GFP and PDGFRα. The density of PDGFRα^+^ cells within the LES and esophagus was greater than that of ICC-IM and two distinct populations of PDGFRα^+^ cells, submucosal (PDGFRα-SM) and intramuscular (PDGFRα-IM), were observed (Fig. 3g–i). PDGFRα-IM occupied a similar anatomical niche to ICC-IM and likewise were found within both smooth and skeletal muscle regions (Fig. 3g–i). Unlike ICC-IM, the density of submucosal and intramuscular PDGFRα^+^ cells was consistent throughout the LES and esophagus. Additional dual-labeling IHC studies were performed on whole-mount preparations of the LES clasp and esophagus to evaluate the expression of SK3. PDGFRα-IM in other regions of the GI tract express SK3 (Cobine et al., 2018, Hannigan et al., 2024, Iino and Nojyo, 2009, Kurahashi et al., 2011, Peri et al., 2013) and this channel has been shown to underlie the hyperpolarization in response to purines released from enteric neurons. PDGFRα-SM were highly branched stellate-shaped cells that formed a dense network, whereas PDGFRα-IM were more spindle-shaped in appearance (Supplemental Fig. 2). SK3 was expressed in PDGFRα-IM but not PDGFRα-SM (Fig. 4bi–iv, Supplemental Fig. 3) suggesting that these two populations likely have differing functional roles in this region. The highest degree of colocalization between SK3 and PDGFRα was observed in the LES clasp (Fig. 4bi). Although some PDGFRα-IM in the esophagus expressed SK3, most of these cells did not, suggesting potential differences in their functional role between regions (Fig. 4bii–iv).

### Relationship of ICC-IM and PDGFRα-IM

Dual labeling IHC was performed on whole-mount preparations of LES clasp and esophagus using antibodies against ANO1 and PDGFRα to determine the relationship of ICC and PDGFRα^+^ cells. ICC-IM and PDGFRα-IM ran parallel to one another and to smooth and skeletal muscle cells, and often made contact with one another (Fig. 4ci–iv). This suggests that ICC-IM and PDGFRα-IM in the mouse LES and esophagus may communicate with one another as well as with SMCs and skeletal muscle.

### Distribution of inhibitory motor nerve fibers

**Figure 5:**
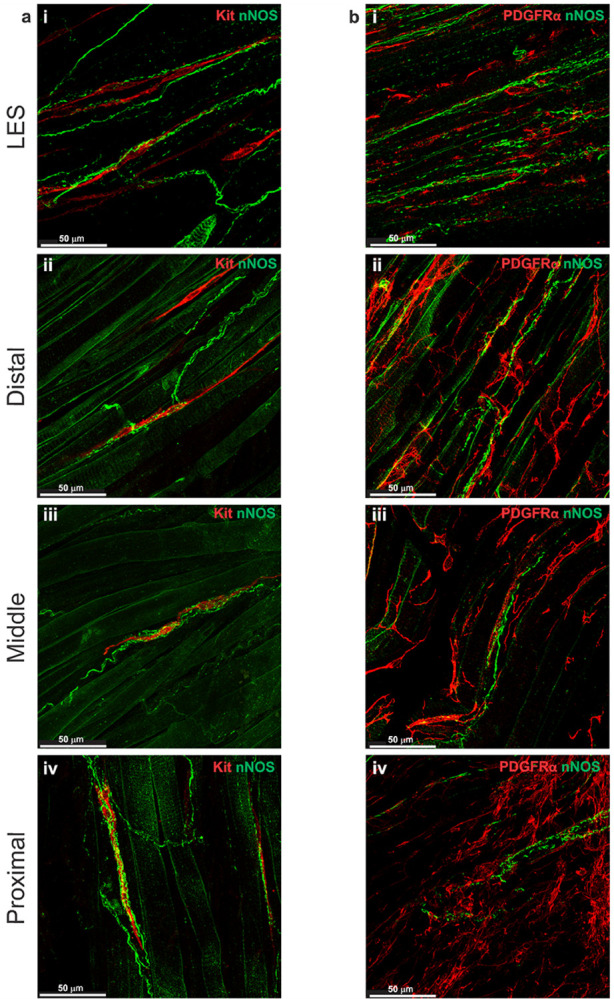
Nitrergic nerve fibers are closely associated with ICC-IM and PDGFRα-IM IHC labeling of whole-mount LES and esophagus with a neuronal nitric oxide synthase (nNOS) antibody (green) demonstrates the presence of nitrergic neurons within smooth muscle and around skeletal muscle regions throughout the LES and esophagus **a-b**. **a** Dual IHC labeling using nNOS (green) and Kit (red) revealed that ICC-IM are closely associated with nitrergic nerve fibers in the LES clasp (i), distal (ii), middle (iii) and proximal esophagus (iv). **b** shows similar intimate associations between nNOS^+^ nerve fibers (green) and PDGFRα-IM (red) in the LES clasp (i) and throughout the esophagus (ii-iv).

**Figure 6:**
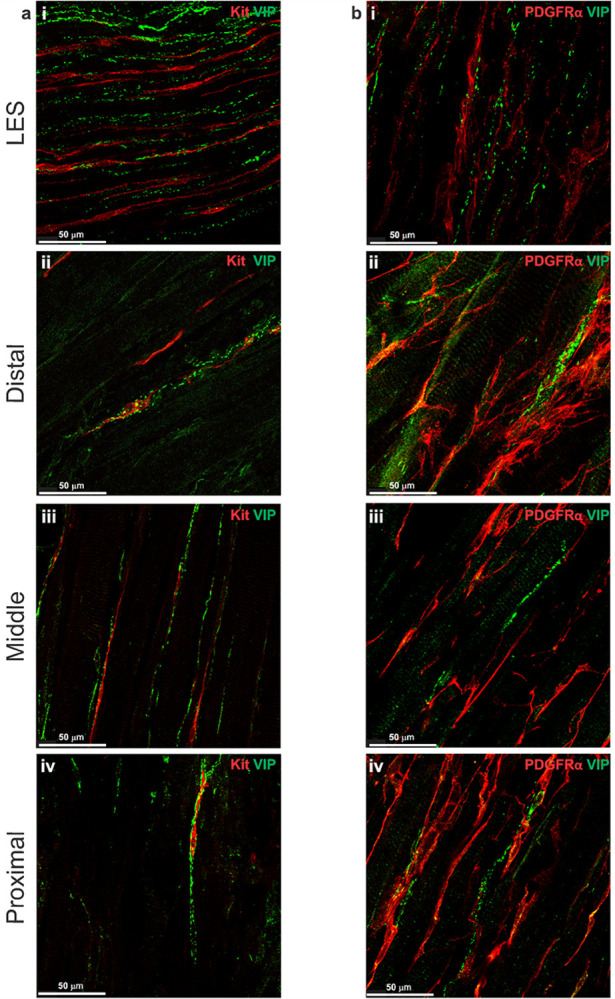
VIPergic nerve fibers are closely associated with ICC-IM and PDGFRα-IM IHC labeling in the mouse whole-mount LES and esophagus with an antibody for vasoactive intestinal peptide (VIP, green) demonstrates the presence of VIP^+^ nerve fibers within smooth muscle and around skeletal muscle bundles **a-b**. **a** Dual IHC labeling for VIP (green) and Kit (red) revealed that ICC-IM are closely associated with VIP^+^ nerve fibers in the LES clasp (i), distal (ii), middle (iii) and proximal esophagus (iv). **b** shows similar associations between VIP^+^ nerve fibers (green) and PDGFRα-IM (red) in the LES clasp (i) and throughout the esophagus (ii-iv).

Nitrergic and VIPergic motor nerves were examined in whole-mount preparations of LES clasp and esophagus using antibodies against nNOS and vasoactive intestinal peptide (VIP) respectively. No distinct myenteric plexus was observed in our studies therefore, we focused on examining neuronal projections within the muscle. The LES clasp had the highest density of both nNOS^+^ and VIP^+^ nerves which formed long, punctate projections that ran in the direction of the CM (Fig. 5ai, bi, 6ai, bi). nNOS^+^ and VIP^+^ nerve fibers decreased in density in the esophagus, and were found within and between smooth muscle bundles (Fig. 5aii, bii, Fig. 6aii, bii). Additionally, nNOS^+^ and VIP^+^ fibers occupied the spaces between skeletal muscle bundles (Fig. 5aii–iv, bii–iv, Fig. 6aii–iv, bii–iv). In comparison to the LES, nNOS^+^ and VIP^+^ nerve fibers in the muscular layer of the esophagus had shorter, spiraling projections. The expression of VIP^+^ nerve fibers throughout the LES clasp and esophagus was similar to that of nNOS^+^ neurons, likely due to reported colocalization of NO and VIP within the same motor nerves (Keef et al., 2013).

### Relationship of inhibitory motor nerve fibers to ICC-IM

Dual IHC labeling of nNOS and Kit or VIP and Kit in whole-mount LES and esophagus preparations revealed that nNOS^+^ and VIP^+^ nerve fibers and ICC-IM ran parallel to the CM in the LES clasp and that ICC-IM formed close contacts with nitrergic and VIPergic nerves (Fig. 5ai, 6ai). This is in alignment with previous studies that reported a close association between ICC and nitrergic neurons in other GI tissues (Blair et al., 2012, Burns et al., 1996, Cobine et al., 2011, Wang et al., 2000) suggesting that ICC-IM play a role in mediating this pathway. Where ICC-IM and nNOS^+^ nerve fibers were closely associated in the esophagus, ICC-IM were enveloped by nNOS^+^ fibers in a woven pattern (Fig. 5aii–iv). VIP^+^ nerve fibers made similar woven patterns where they came in close proximity to ICC-IM (Fig. 6aii–iv).

### Relationship of inhibitory motor nerve fibers to PDGFRα-IM

The association between inhibitory nerves and PDGFRα-IM was examined using dual IHC labeling of nNOS and PDGFRα or VIP and PDGFRα in whole-mount preparations. nNOS^+^ and VIP^+^ nerve fibers ran parallel to PDGFRα-IM in the LES clasp and cell bodies of PDGFRα-IM occasionally came in close proximity to axonal projections of nerve fibers (Fig. 5bi, Fig. 6bi). This was true in the distal, middle and proximal esophagus where the large population of PDGFRα-IM frequently associated with VIP^+^ and nNOS^+^ neurons (Fig. 5bii–iv, Fig. 6bii–iv).

### Distribution of cholinergic and adrenergic motor nerve fibers

**Figure 7:**
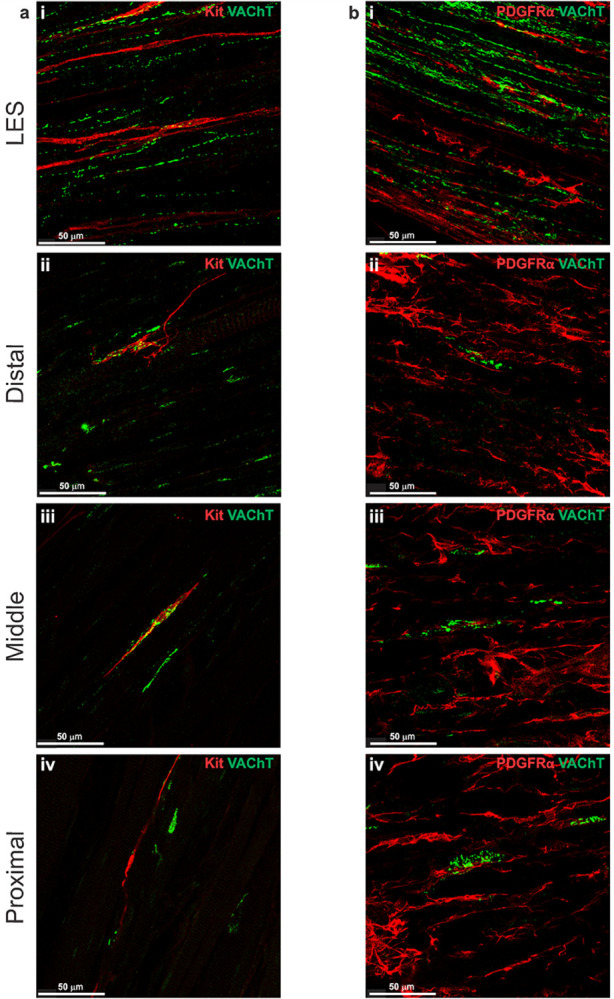
Cholinergic nerve fibers are closely associated with ICC-IM and PDGFRα-IM IHC labeling in the mouse whole-mount LES and esophagus using an antibody against vesicular acetylcholine transporter (VAChT, green). Panels **a-b** show VAChT^+^ nerve fibers in the LES clasp (i) and throughout the esophagus (ii-iv). **a** Co-labeling of the same preparations with Kit (red) demonstrated very close associations between cholinergic nerve fibers (green) and ICC-IM (red) in the mouse LES clasp (i), distal (ii), middle (iii) and proximal esophagus (iv). **b** Co-labeling of VAChT (green) and PDGFRα (red) revealed that PDGFRα-IM also formed close contacts with cholinergic neurons in the LES clasp (i), and throughout the esophagus (ii-iv).

**Figure 8:**
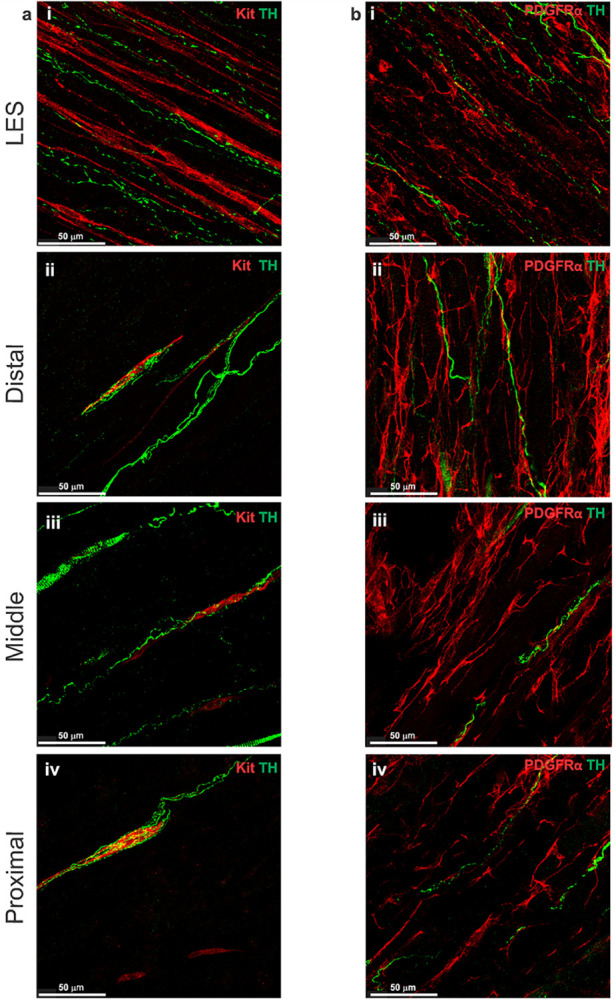
Adrenergic nerve fibers are closely associated with ICC-IM and PDGFRα-IM Tyrosine hydroxylase (TH) antibody labeling was used to identify adrenergic nerve fibers in whole-mount mouse LES and esophagus. Panels **a-b** show the presence of TH^+^ nerve fibers (green) in the LES clasp (i) and throughout the esophagus (ii-iv). **a** Co-labeling with Kit (red) demonstrated close associations between TH^+^ nerve fibers and ICC-IM in the mouse LES clasp (i), distal (ii), middle (iii) and proximal esophagus (iv). **b** Co-labeling of TH (green) and PDGFRα (red) revealed that PDGFRα-IM also made contact with adrenergic neurons in the LES clasp (i), and throughout the esophagus (ii-iv).

Cholinergic and adrenergic motor nerves were examined in whole-mount preparations of LES clasp and esophagus using antibodies against vesicular acetylcholine transporter (VAChT) and tyrosine hydroxylase (TH) respectively. In the LES, VAChT^+^ and TH^+^ nerve fibers ran parallel to the CM and formed long axonal projections with a punctate appearance (Fig. 7ai, bi, Fig. 8ai, bi). In the distal, middle and proximal esophagus the number of VAChT^+^ and TH^+^ nerve fibers was lower than in the LES, however due to the vesicular target of our VAChT antibody, labeling was restricted to motor end plate structures (Fig. 7aii–iv, bii–iv, Fig. 8aii–iv, bii–iv). VAChT^+^ and TH^+^ nerve fibers were observed within and between smooth muscle bundles, and between skeletal muscle bundles. VAChT^+^ motor end plate labeling was only visible within the skeletal muscle bundles. TH^+^ labeling in the esophagus varied in appearance; some nerve fibers had long twirling projections (Fig. 8bii), whereas others were more spiraled in appearance (Fig. 8aiv).

### Relationship of cholinergic and adrenergic motor nerve fibers to ICC-IM

Dual labeling IHC of VAChT and Kit or TH and Kit was carried out to examine the relationship of cholinergic and adrenergic nerves to ICC-IM. These studies revealed that VAChT^+^ and TH^+^ nerve fibers ran parallel to ICC-IM in the LES clasp, and that both nerve fiber types made frequent contacts with ICC-IM (Fig. 7ai, Fig. 8ai). This corroborates previous studies showing that ICC play a role in modulating cholinergic neurotransmission in the GI tract (Sung et al., 2018, Ward et al., 2000). In the esophagus where VAChT^+^ and TH^+^ nerve fibers interacted with ICC-IM, they were woven in nature and appeared to envelope the ICC-IM (Fig. 7aii–iv, Fig. 8aii–iv).

### Relationship of cholinergic and adrenergic motor nerve fibers to PDGFRα-IM

Dual labeling IHC of VAChT and PDGFRα or TH and PDGFRα revealed that VAChT^+^ and TH^+^ nerve fibers ran in the same orientation as PDGFRα-IM in the LES clasp and that PDGFRα-IM frequently formed close associations with both cholinergic and adrenergic nerves (Fig. 7bi, Fig. 8bi). In the esophagus PDGFRα-IM often associated with VAChT^+^ motor end plate labeling and TH^+^ projections (Fig. 7bii–iv, Fig. 8bii–iv). Interestingly PDGFRα-IM were less intimately associated with VAChT^+^ and TH^+^ nerves than ICC, despite the higher number of PDGFRα-IM present throughout the muscle.

### Relationship of enteric GFAP^+^ cells to ICC-IM and PDGFRα-IM

**Figure 9:**
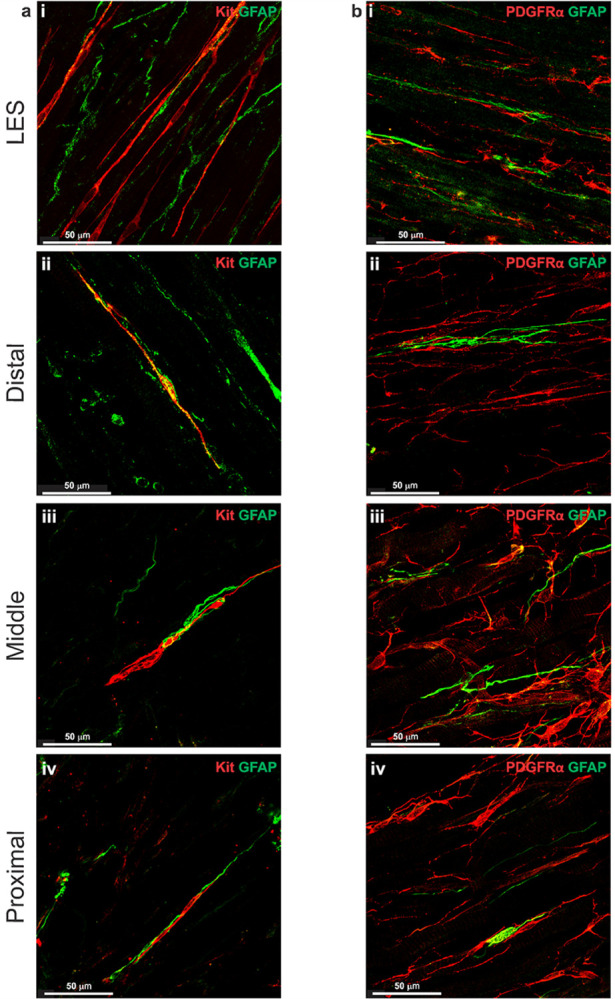
GFAP+ cells form close contacts with ICC-IM and PDGFRα-IM **a** Co-labeling of whole-mount mouse esophagus and LES with antibodies against Kit (red) and glial fibrillary acidic protein (GFAP, green) revealed that ICC-IM make contact with GFAP^+^ cells in the LES (i) and esophagus (ii-iv). **b** Co-labeling of whole-mount mouse LES and esophagus tissues antibodies against PDGFRα (red) and GFAP (green) revealed that PDGFRα-IM also formed close contacts with GFAP^+^ cells in the LES clasp (i), and esophagus (ii-iv). GFAP^+^ cells were found within muscle bundles and varied in morphology, from spindle-shaped cells with distinct cell bodies and long projections (ai-aiv, bi, biii) to clusters of branched cells that appeared to form basket-like-structures (bii, biv).

Enteric glia have previously been shown to influence the activity of enteric neurons (Seguella and Gulbransen, 2021, Thomasi and Gulbransen, 2023). Therefore, we also examined the distribution of glia within the LES clasp and esophagus and their relationship to interstitial cells. Whole-mount preparations were dual labelled with an antibody against glial fibrillary acidic protein (GFAP) and antibodies for Kit or PDGFRα. GFAP^+^ cells were identified in the CM in the LES and within skeletal muscle throughout the esophagus. In the LES clasp, GFAP^+^ cells were spindle-shaped with prominent cell bodies and ran in the direction of the CM without forming a distinct plexus (Fig. 9ai, bi). GFAP^+^ cells were closely associated with ICC-IM and PDGFRα-IM (Fig. 9ai, bi). In the esophagus, the morphology of GFAP^+^ cells varied from spindle-shaped with visible cell bodies (Fig. 9aii, aiv), to cells with long spiraling projections (Fig. 9aiii, biii). Esophageal GFAP^+^ cells occasionally were found in close proximity to ICC-IM and PDGFRα-IM (Fig. 9aii–iv, bii–iv).

## Discussion

ICC and PDGFRα^+^ cells form an electrical syncytium with SMCs known as the SIP syncytium (Sanders, et al., 2024, Sanders, et al., 2014). ICC generate electrical slow waves which conduct to SMCs via gap junctions (Alberti et al., 2007, Huizinga et al., 1995, Sanders et al., 2006, Sanders et al., 2014, Ward et al., 1994). Additionally, ICC and PDGFRα^+^ cells have roles as mediators of neuromuscular transmission (NMT) (Baker et al., 2018a, Baker et al., 2018b, Baker et al., 2013, Baker et al., 2015, Burns et al., 996, Cobine et al., 2018, Cobine et al., 2014, Hannigan et al., 2024, Hwang et al., 2022, Kurahashi et al., 2022, Kurahashi et al., 2020, Kurahashi et al., 2011, Sung et al., 2018, Ward et al., 2000). Thus, these cells play an important role in the regulation of GI motility.

Motility patterns differ between the LES and the esophagus. In the mouse, the LES generates tone ((Drumm et al., 2022) and current study) whereas the distal esophagus exhibits rhythmic phasic contractions and little tone (current study). This is in alignment with studies in the dog where the distal LES generated tone but the proximal LES had rhythmic phasic contractions (Huizinga and Walton, 1989). The dog esophagus has similar anatomical structure to the mouse in that it is composed predominantly of skeletal muscle (Allescher et al., 1988, Sang and Young, 1997), despite this, electric slow waves have been recorded from the dog proximal LES and are thought to underlie phasic contractile activity (Huizinga and Walton, 1989). Therefore, similar physiology may be observed in the mouse esophagus. As ICC and PDGFRα^+^ cells regulate SMC activity in other GI regions, the aims of the current study were to better evaluate their distribution and relationship to one another and to inhibitory and excitatory motor neurons and to determine if the mouse represents a viable model for studying esophageal motility.

### Distribution of skeletal and smooth muscles in the mouse esophagus

Using two different approaches, the distributions of SMCs and skeletal muscle cells were evaluated. In sections of the mouse stomach and esophagus stained with Masson’s trichrome, it was noted that SMCs were arranged in distinct bundles separated by connective tissue septa within the LES. Similarly, in sections of the GEJ taken from a mouse expressing eGFP in SMCs (SmMHC^eGFP/+^), bundles of smooth muscle were apparent in the LES. From both approaches it was clear that there was a thickening of the CM layer consistent with the anatomy of other GI sphincters such as the internal anal sphincter (IAS) (Hall et al., 2014). A distinct band of smooth muscle was observed just beneath the mucosal layer that traversed the entire length of the esophagus. This is consistent with the location of muscularis mucosae (Rishniw et al., 2007).

By labeling sections of the SmMHC^eGFP/+^ mouse with an antibody targeting skeletal muscle myosin heavy chain it was apparent that SMCs declined in density in the proximal direction. It has previously been suggested that the only SMCs present within the mouse esophagus are those just caudal to the LES (Rishniw et al., 2007). In the present study, SMCs were observed within the GEJ, with the distal esophagus in this region being composed of SMCs and skeletal muscle cells in a 1:3 ratio. However, SMCs were present within the muscularis externa throughout the length of the esophagus though their density declined significantly in the middle and proximal regions. Given that the GEJ had both SMCs and skeletal muscle cells, it suggests that this region is more similar to the transitional zone in the human esophagus where both smooth and skeletal muscle is located (Faussone-Pellegrini and Cortesini, 1986, Meyer et al., 1986, Sang and Young, 1997) and that perhaps the anatomy of the mouse esophagus may be closer to that of humans than thought initially.

### Distribution of interstitial cells

ICC-IM were distributed throughout the LES and were greatest in density within this region. No myenteric ICC population was noted in the LES. These findings are in agreement with previous studies evaluating the distribution of ICC in the LES (Ward et al., 1998). ICC-IM were abundant in the distal esophagus within the GEJ where they were closely aligned with SMCs. These observations are in keeping with previous studies in the dog LES where ICC were most abundant in the most proximal aspect of the LES (Berezin et al., 1994). It was previously postulated that ICC may be responsible for the generation of rhythmic electrical activity in the proximal LES of the dog (Huizinga and Walton, 1989). This aligns with the rhythmic whole-cell Ca^2+^ transients observed in ICC-IM within the mouse GEJ (Hannigan et al., 2023) where phasic contractions are present. Similar Ca^2+^ activity has been described for ICC-IM in the mouse IAS where these cells have been suggested to underlie slow wave generation (Cobine et al., 2017, Hall et al., 2014, Hannigan et al., 2020).

ICC-IM throughout the GI tract including in the mouse LES and Cynomolgus monkey LES and distal esophagus express ANO1, a conductance that is required for pacemaker activity (Cobine et al., 2017, Drumm et al., 2022, Gomez-Pinilla et al., 2009, Hwang et al., 2009). In the present study, the greatest density of ANO1^+^ cells was in the LES and GEJ. All cells that expressed ANO1 were Kit^+^, indicating that all ICC-IM express ANO1. Surprisingly, even ICC-IM associated with skeletal muscle bundles in the middle and proximal esophagus expressed ANO1. It is unclear why ANO1^+^ ICC-IM are associated with skeletal muscle however this poses an interesting question and their role in this more proximal region surely warrants further investigation.

A second population of interstitial cells known as PDGFRα^+^ cells are found throughout the GI tract (Blair et al., 2012, Cobine et al., 2011, Cobine et al., 2018, Iino et al., 2009a, Iino and Nojyo, 2009, Kurahashi et al., 2012, Kurahashi et al., 2011). These cells are distinct from ICC though occupy a similar anatomical niche (Blair et al., 2012, Cobine et al., 2011, Iino et al., 2009a, Kurahashi et al., 2011). Before it was known that these cells express PDGFRα, they were simply referred to as “fibroblast-like cells” (Iino et al., 2009a). Cells called “telocytes” with ultrastructure distinct from ICC, were found in electron microscopy studies of the rat esophagus (Rusu et al., 2012). These cells are likely to be PDGFRα^+^ cells based on their morphology and the fact that telocytes express PDGFRα in the GI tract (Vannucchi et al., 2013).

Studies utilizing electron microscopy demonstrated that interstitial cells with differing characteristics are present within the esophagus of the mouse, dog, cat, monkey, opossum and human (Allescher et al., 1988, Berezin et al., 1987, Berezin et al., 1994, Daniel and Posey-Daniel, 1984, Farre et al., 2007, Faussone-Pellegrini and Cortesini, 1985, Faussone-Pellegrini et al., 2013, Huizinga et al., 2008, Rumessen et al., 2001, Wong et al., 1990). In electron microscopy, ICC are distinguished from PDGFRα^+^ cells based on the presence of caveolae, an abundance of mitochondria and well-developed Golgi apparatus and endoplasmic reticulum (Komuro et al., 1999). Fibroblasts or “fibroblast-like cells” have been observed in studies of the esophagus (Farre et al., 2007, Rumessen et al., 2001), but little attention was given to these cells as nothing was known of their role as regulators of motility at the time of these studies. However, just as in other regions of the GI tract (Cipriani et al., 2011, Horiguchi and Komuro, 2000), ICC and PDGFRα^+^ cells formed gap junctions with SMCs and were closely associated with nerve varicosities (Allescher et al., 1988, Berezin et al., 1987, Berezin et al., 1994, Daniel and Posey-Daniel, 1984, Farre et al., 2007).

Different populations of PDGFRα^+^ cells have now been described throughout the GI tract, including but not limited to, populations in the submucosa, plane of the myenteric plexus and within muscle bundles (i.e., intramuscular) (Cobine et al., 2011, Iino, et al., 2009a). In the present study, PDGFRα^+^ cells were found throughout the musculature as well as in the submucosal region. PDGFRα-IM were distinct from ICC-IM but were closely associated with ICC-IM throughout the LES and esophagus. This is in keeping with human esophageal studies where ICC-IM and PDGFRα-IM were shown to be closely associated in healthy controls and achalasic patients (Chen et al., 2013). Unlike ICC or SMCs, no dramatic decline in the density of PDGFRα-IM was observed in the more proximal regions of the mouse esophagus. These cells were once again closely associated with SMCs in the LES and GEJ and as described previously (Chen et al., 2013, Iino et al., 2009a), they persisted in skeletal muscle regions of the esophagus.

### Relationship of ICC and PDGFRα^+^ cells to motor nerve fibers

In order for ICC-IM and PDGFRα-IM to mediate neural responses they must occupy a similar anatomical niche to motor nerve fibers. Both interstitial cell types have been shown to be in close apposition to nNOS^+^ nerve fibers throughout the mouse and Cynomolgus monkey GI tract (Blair et al., 2012, Cobine et al., 2010, Cobine et al., 2011, Cobine et al., 2018, Kurahashi et al., 2012, Wang et al., 1999, Wang et al., 2000) and ICC-IM have also been shown to be closed associated with TH^+^ nerve fibers in the Cynomolgus monkey rectum (Cobine et al., 2010). ICC-IM and PDGFRα-IM are also located close to nerve varicosities in the rat LES (Farre et al., 2007) and to nNOS^+^ nerves in the human esophagus (Chen et al., 2013). Additionally, ICC-IM and PDGFRα-IM in various GI regions express sGC, PKG and IRAG, downstream mediators of nitrergic NMT (Baker et al., 2018a, Cobine et al., 2014, Hannigan et al., 2024, Iino et al., 2008, Iino et al., 2009b). In the present study, ICC-IM and PDGFRα-IM were in close alignment with intrinsic motor neurons immunopositive for nNOS, VIP and VAChT as well as TH^+^ neurons in the LES and esophagus. These data are consistent with previous immunohistochemical studies demonstrating a close association between ICC-IM and nitrergic, VIPergic, cholinergic and adrenergic nerve fibers (Beckett et al., 2005, Blair et al., 2012, Cobine et al., 2011, Cobine et al., 2018, Keef et al., 2013, Kurahashi et al., 2012) and between PDGFRα^+^ cells and nNOS^+^ nerves in the mouse IAS, Cynomolgus monkey LES and IAS and human esophagus (Blair et al., 2012, Chen et al., 2013, Cobine et al., 2010, Cobine et al., 2011).

PDGFRα^+^ cells mediate purinergic NMT in the mouse stomach, colon and IAS (Baker et al., 2013, Baker et al., 2015, Cobine et al., 2018, Hannigan et al., 2024, Kurahashi et al., 2014, Kurahashi et al., 2011, Peri et al., 2013). More recently these cells have also been shown to have a role in responses to PACAP and catecholamines in the colon (Kurahashi et al., 2022, Kurahashi et al., 2020). Responses to purines, catecholamines and PACAP all involve the activation of SK3 channels expressed in PDGFRα^+^ cells (Cobine et al., 2018, Hannigan et al., 2024, Iino and Nojyo, 2009, Kurahashi et al., 2011, Peri et al., 2013). PDGFRα^+^ cells also express P2Y_1_R, a1 adrenoceptors and PAC_1_R (Baker et al., 2013, Hannigan et al., 2024, Kurahashi et al., 2022, Kurahashi et al., 2020, Peri et al., 2013) in addition to SK3. Therefore, to determine whether PDGFRα^+^ cells in the mouse esophagus have the ability to mediate similar neural responses, the expression of SK3 was evaluated. SK3 was observed in PDGFRα-IM within the LES and GEJ but not in PDGFRα-SM. Weak SK3 labeling was noted in structures resembling vasculature in esophagus but not LES, consistent with previous observations that endothelial cells express SK3 (Peixoto-Neves et al., 2023).

These data suggest that PDGFRα-IM have the capacity to mediate neural responses in the distal esophagus and LES. However, the role of purines in this region is unclear. Though apamin-sensitive inhibitory junction potentials (IJPs) have been described in the mouse LES clasp muscle previously (Zhang et al., 2009, Zhang et al., 2008), studies in the human esophageal body, LES clasp and sling muscles have demonstrated an absence of purinergic IJPs (Lecea et al., 2011). In the human and Cynomolgus monkey IAS, purinergic inhibitory responses are absent just as described for the human esophagus and LES muscles (Cobine et al., 2018, Lecea et al., 2011, O’Kelly et al., 1993). Despite the lack of purinergic response, SK3 was expressed on PDGFRα^+^ cells in the Cynomolgus monkey IAS (Cobine et al., 2018) where sympathetic inputs are excitatory to the muscle (Cobine et al., 2010). Thus, the expression of SK3 does not necessarily correlate to purinergic signaling and may instead relate to other pathways such as adrenergic or PACAP. Given the potential function of SK3 in various neuromuscular pathways, greater exploration of the role of these channels in PDGFRα^+^ cells within the esophagus and LES is warranted. Additionally, further investigation of the role of PDGFRα-SM and SK3-negative PDGFRα-IM within the mouse esophagus is required to determine the function of PDGFRα^+^ cells in this region.

### Distribution of glia and their anatomical relationship to interstitial cells

At least six glial populations are found throughout the colon and small intestine (Seguella and Gulbransen, 2021). These include intra-ganglionic populations in the myenteric and submucosal regions and extra-ganglionic populations which include but are not limited to, intramuscular glia (Seguella and Gulbransen, 2021). Though the roles of various populations of enteric glia are being uncovered in the colon and small intestine, little is known about these cells in the other GI regions. Previous studies in the human esophagus demonstrated the presence of S100β^+^ cells (Hoshino et al., 2013, Qian et al., 2023). S100P is expressed by mature astrocytes within the central nervous system and is also used as a marker for enteric glial cells (Grundmann et al., 2019). To our knowledge, the relationship of glial cells and interstitial cells has never been evaluated in the esophagus or in any region of the GI tract. Thus, to determine the distribution of glia within the esophagus, tissues were labeled with an antibody against GFAP, another marker for astrocytes and enteric glial cells (Grundmann et al., 2019). Intramuscular GFAP^+^ cells were spindle-shaped and largely devoid of branched processes. This morphology is in agreement with what has been demonstrated previously in the intestine (Seguella and Gulbransen, 2021). In the present study, GFAP^+^ cells interacted with both ICC-IM and PDGFRα-IM in the LES and esophagus. It is interesting to note that studies in the colon demonstrated expression of proteolipid protein 1 (PLP-1) in intramuscular glia but not GFAP expression (Rao et al., 2015). Since GFAP is known to be expressed by other cells that modulate neuronal activity in the central nervous system (Jessen et al., 1984) it is plausible that the GFAP^+^ cells seen in the present study may differ from the enteric glial cells described in the intestine. However, their spatial relationship to ICC-IM and PDGFRα-IM suggests that they may have a functional relationship in the esophageal region, particularly as they occupy the space where nerve fibers are located. Further characterization of these cells is therefore warranted.

In summary, here we evaluated the distribution of SMCs, ICC, PDGFRα^+^ cells and motor nerve fibers in the mouse esophagus and LES. We found that the distal esophagus more closely resembles the transition zone between skeletal and smooth muscle in humans. We also found that ICC-IM express ANO1 throughout the esophagus and that these cells are greatest in density within the LES and distal esophagus at the GEJ. PDGFRα^+^ cells were closely associated with ICC-IM and expressed SK3 in the LES and distal esophagus. Both types of interstitial cell were associated with nNOS^+^, VIP^+^, VAChT^+^ and TH^+^ nerve fibers as well as GFAP^+^ cells.

This study provides evidence that the mouse esophagus is not just skeletal muscle in nature and may be more anatomically similar to the human esophagus than thought previously. Additionally, these morphological findings provide a foundation for future functional studies including Ca^2+^ imaging in ICC-IM and SMCs within the GEJ. Future studies will better assess the role of SIP cells in regulating the motility of the esophagus and in mediating responses to inhibitory and excitatory neural inputs. Despite the apparent differences in the makeup of the esophagus between humans and animal models, such as the mouse, studies completed in the mouse likely still have significant relevance for the study of human disease.

## Figures and Tables

**Table 1:** Antibody details.

Primary antibody	Product code/RRID	Source	Monoclonal or polyclonal	Host	Working dilution	Secondary antibody	Working dilution
Anti-GFP	AB13970 RRID:AB_300798	Abcam, Cambridge, Massachusetts, USA.	Polyclonal	Chicken	**1 in 1000**	Alexa Fluor anti-chicken 488, Invitrogen, Carlsbad, CA, USA	**1 in 1000**
Anti-h/mSCFR	AF1356 RRID:AB_354750	R&D Systems, Minneapolis, Minnesota, USA.	Polyclonal	Goat	**1 in 100**	Alexa Fluor anti-goat 594, Invitrogen, Carlsbad, CA, USA	**1 in 1000**
Anti-ANO1	AB53212 RRID:AB_883075	Abcam, Cambridge, Massachusetts, USA.	Polyclonal	Rabbit	**1 in 500**	Alexa Fluor anti-rabbit 594, Invitrogen, Carlsbad, CA, USA	**1 in 1000**
Anti-mPDGFRa	AF1062 RRID:AB_2236897	R&D Systems, Minneapolis, Minnesota, USA.	Polyclonal	Goat	**1 in 100**	Alexa Fluor anti-goat 594, Invitrogen, Carlsbad, CA, USA	**1 in 1000**
Anti-nNOS	SC-648 RRID:AB_630935	Santa Cruz Biotechnology Inc, Dallas, TX, USA	Polyclonal	Rabbit	**1 in 200**	Alexa Fluor anti-rabbit 488, Invitrogen, Carlsbad, CA, USA	**1 in 1000**
Anti-VAChT	AB1588 RRID:AB_2187981	EMD Millipore sigma, Merk KGaA, Darmstadt, Germany	Polyclonal	Guinea pig	**1 in 1000**	Alexa Fluor anti-guineapig 488, Invitrogen, Carlsbad, CA, USA	**1 in 1000**
Anti-VIP	20077 RRID:AB_572270	Immunostar antibodies, Hudson, WI, USA	Monoclonal	Rabbit	**1 in 500**	Alexa Fluor anti-rabbit 488, Invitrogen, Carlsbad, CA, USA	**1 in 1000**
Anti-TH	AB152 RRID:AB_390204	EMD Millipore sigma, Merk KGaA, Darmstadt, Germany	Polyclonal	Rabbit	**1 in 1000**	Alexa Fluor anti-rabbit 488, Invitrogen, Carlsbad, CA, USA	**1 in 1000**
Anti-GFAP	G9269 RRID:AB_477035	EMD Millipore sigma, Merk KGaA, Darmstadt, Germany	Polyclonal	Rabbit	**1 in 500**	Alexa Fluor anti-rabbit 488, Invitrogen, Carlsbad, CA, USA	**1 in 1000**
Anti-SK3	APC-025 RRID:AB_2040130	Alomone Labs, Jerusalem, Israel	Monoclonal	Rabbit	**1 in 1000**	Alexa Fluor anti-rabbit 488, Invitrogen, Carlsbad, CA, USA	**1 in 1000**
Anti-MYH1E (MF20)	AB_2147781 RRID:AB_3099659	Deposited to the DSHB by Fischman, D.A. (DSHB Hybridoma Product MF 20)	Monoclonal	Mouse	**1 in 200**	Alexa Fluor anti-mouse 594, Invitrogen, Carlsbad, CA, USA	**1 in 1000**

**Table 2:** Ex vivo muscle thickness measurements and in situ esophageal measurements

a. Measurements taken for muscle thickness.						
	LES	Distal	Middle	Proximal	Muscle bundles	Panel
CM	634.7	168.4	93.0	131.9	5.0	2C
	695.9	224.3	139.6	101.0	7.0	2B
	527.8	186.9	98.0	95.5	4.0	2A
**AVERAGE**	**619.5**	**193.2**	**110.2**	**109.5**	**5.3**	
**STDEV**	**69.5**	**23.3**	**20.9**	**16.0**		
LM	N/A	133.9	76.4	99.3	N/A	2C
	189.4	112.1	102.8	89.7	N/A	2B
	N/A	145.2	87.9	50.3	N/A	2A
**AVERAGE**	**189.4**	**130.4**	**89.0**	**79.8**		
**STDEV**	**0.0**	**13.7**	**10.8**	**21.2**		
b. In vivo length of intact esophagus						
DOB	sex	length (mm)				
23-Sep	M	32.0				
23-Sep	M	35.0				
1-Nov-23	F	31.0				
1-Nov-23	F	34.0				
31-Oct-23	M	32.0				
31-Oct-23	M	33.0				
	**Average**	32.8				
	**stdev**	1.3				

## Data Availability

Reasonable requests for data are available from the corresponding author.
